# New developments in the pathology of malignant lymphoma. A review of the literature published from September 2015–December 2015

**DOI:** 10.1007/s12308-016-0269-4

**Published:** 2016-02-22

**Authors:** J. Han van Krieken

**Affiliations:** Department of Pathology, Radboud University Medical Centre, P.O. Box 9101, 6500 HB Nijmegen, The Netherlands

## Introduction

It remains a challenge to discuss every 3 months the published literature on the pathology of lymphomas. A pleasant challenge, because I need to read many interesting works, but also an impossible one: I cannot read everything. Nevertheless, I hope that also this month, I can provide the readership with an interesting summary of my personal selection.

## Biology of lymphoma

### Hodgkin lymphoma

CD30 expression is a hallmark of Hodgkin lymphoma (HL), and this is associated with activation of the nuclear factor-κB (NF-κB) pathway. Epstein-Barr virus (EBV) latent membrane protein-1 (LMP-1) and ligand-independent signaling by overexpressed CD30 are both known to cause activation of NF-κB in lymphomas, but in normal cells hyperactivation of NF-κB triggers cellular senescence and apoptosis. Ishikawa et al. [1] show that IκB-ζ, an inducible regulator of NF-κB, is constitutively expressed in Burkitt lymphoma (BL) and HL cell lines. In addition, immunohistochemically nuclear IκB-ζ was positive in BL cells and Hodgkin and Reed-Sternberg (HRS) cells. Expression of LMP-1 and CD30 increased IκB-ζ expression at the transcriptional level. IκB-ζ promoter was regulated by activation of the NF-κB-inducing kinase (NIK)/IκB kinase/NF-κB pathway via the carboxyl-terminal tumor necrosis factor (TNF) receptor-associated factor (TRAF)-interacting regions of LMP-1 and CD30. Interestingly, IκB-ζ inhibited NF-κB activation by LMP-1 and CD30. The results suggest that NF-κB-induced IκB-ζ negatively modulates NF-κB hyperactivation, resulting in a fine balance that ultimately endows a net evolutionary benefit to the survival of BL and HL cells. This work shows how complicated checks and balances are in normal and neoplastic cells and how difficult it is to deduct function from mere immunohistochemical analysis of tumors.

Another example is provided by Oelmann et al. [2] who studied expression of IL-1beta, IL-1R1, and IL-1R2 in 17 HL by mRNA in situ hybridization (ISH). Signaling through the IL-1-receptor type 1 (IL-1R1), IL-1 is required for initiation and maintenance of diverse activities of the immune system, and a second receptor, IL-1R2, blocks IL-1 signal transduction. IL-1beta expressing cells, morphologically consistent with endothelial cells and fibroblasts, occurred in all HL tissues with elevated transcript levels in areas of active fibrosis. HRS cells of all cases expressed low IL-1R1 transcript levels in some tumor cells and high levels of IL-1R2 in large proportions of HRS cells. Only few bystander cells showed low levels of IL-1R1 and IL-1R2 RNA. HL patient sera carried variably amounts of IL-1R2 protein with significantly increased titers in patients with active disease compared to patients in complete remission and control individuals without HL. Western blots and co-immunoprecipitations showed binding of the IL-1R2 to the intracellular IL-1R-accessory protein (IL-1IRAcP). These data suggest functions of the IL-1R2 as a “decoy-receptor” sequestrating paracrine IL-1 extracellularly and intracellularly by engaging IL-1IRAcP, thus depriving IL1-R1 molecules of their extracellular and intracellular ligands. Expression of IL1-R2 by HRS cells seems to contribute to local and systemic modulation of immune function in HL.

### B cell lymphomas

Nowadays, whole genome or exome sequencing (WGS; WES) has become a widely available tool so we can expect rapid increase in the knowledge on genetic changes in many tumors. Peveling-Oberhag et al. [3] investigated splenic marginal zone lymphoma (sMZL) using this technique in two cases. They found 25 different somatic mutations, including known mutations in the *NOTCH2* and *MYD88* genes, but the other 23 had not been associated with SMZL before. However, in none of 24 additional sMZL, these latter mutations were also found. Therefore, this approach was not very successful in finding recurrent genetic alterations in this study, but further work is needed.

The gene encoding the lysine-specific histone methyltransferase KMT2D has emerged as one of the most frequently mutated genes in follicular lymphoma (FL) and diffuse large B cell lymphoma (DLBCL), but the biological consequences of these mutations are not known. Two groups of researchers took on the challenge to determine the role of these mutations in lymphoma development and come with similar results. Ortega-Molina et al. [4] show in mouse models that KMT2D affects methylation of histone H3 and expression of a set of genes, including those in the CD40, JAK-STAT, Toll-like receptor, and B cell receptor signaling pathways. Other KMT2D target genes include frequently mutated tumor suppressor genes such as TNFAIP3, SOCS3, and TNFRSF14. Furthermore, they show that KMT2D functions as a tumor suppressor and that its loss in B cells results in diminished B cell differentiation and class switch recombination as well as lymphoma development. Zhang et al. [5] show that FL- and DLBCL-associated KMT2D mutations impair KMT2D enzymatic activity, leading to diminished global H3K4 methylation in germinal-center (GC) B cells and DLBCL cells. Conditional deletion of KMT2D early during B cell development, but not after initiation of the GC reaction, results in an increase in GC B cells and enhances B cell proliferation in mice. Moreover, genetic ablation of KMT2D in mice overexpressing Bcl2 increases the incidence of GC-derived lymphomas resembling human tumors. These findings suggest that KMT2D acts as a tumor suppressor gene whose early loss facilitates lymphomagenesis by remodeling the epigenetic landscape of the cancer precursor cells.

BL and FL both have features of GC B cells but are biologically and clinically quite distinct. Kretzmer et al. [6] performed whole-genome bisulfite, genome and transcriptome sequencing in 13 immunoglobulin (IG)-MYC translocation-positive BL, 9 BCL2 translocation-positive FL and 4 normal GC B cell samples. There was different methylation of intragenic regions between BL and FL that strongly correlated with expression of associated genes. The results demonstrate a tight connection between somatic mutation, DNA methylation, and transcriptional control in key B cell pathways deregulated differentially in different types of GC B cell lymphomas.

Hartmann et al. [7] tried to gain better understanding of the signaling molecules in the microenvironment of B cell samples, which in the end, might lead to new treatment approaches. They analyzed B-CLL infiltrated lymph nodes from 43 different patients for expression of the chemokine CCL3, Ki-67, macrophages, and T cell subsets by immunohistochemistry. CCL3 expression was detected in 24 of 43 cases (56 %), particularly within the proliferation centers, which was associated with higher numbers of CD3+ T cells and CD57+ cells, and higher proliferation rates. Obviously, more data are required before this type of insight results in specific treatments.

Activated B cell (ABC) DLBCL relies on B cell receptor (BCR) signaling but only a part of patients benefit form BCR pathway inhibition. Young et al. [8] developed a series of ABC cells that differed solely with respect to the immunoglobulin heavy chain variable region (IgH v). The viability of one VH4-34 ABC line and the ability of its BCR to bind to its own cell surface depended autoreactivity to self-glycoproteins. The BCR of another ABC line reacted with self-antigens in apoptotic debris, and the survival of a third ABC line was sustained by reactivity of its BCR to an idiotypic epitope in its own V region. The authors conclude that a diverse set of self-antigens is responsible for maintaining the survival of ABC DLBCL cells. As a therapy, removal of such stimuli seems not feasible, but addressing the BCR is a potential alternative.

### T cell lymphoma

Some lymphomas are closely related to specific environments in the body. An example is the tight relation with infiltration in the epithelium of some cutaneous T cell lymphomas (T-NHL). Adachi et al. [9] investigated normal hair follicles, which are known to contribute to skin dendritic cell homeostasis through chemokine production. They demonstrate that CD4(+) and CD8(+) skin-resident memory T cells (TRM cells), which are responsible for long-term skin immunity, reside predominantly within the hair follicle epithelium of the normal skin. Hair follicle expression of IL-15 was required for CD8(+) TRM cells, and IL-7 for CD8(+) and CD4(+) TRM cells, to exert influx in the hair follicle epithelium. Also in a model of cutaneous T-NHL, epidermotropic CD4(+) TRM lymphoma cell localization depended on the presence of hair follicle-derived IL-7. These findings implicate hair follicle-derived cytokines as regulators of malignant and non-malignant TRM cell tissue residence, and they suggest that the cytokines may be targeted therapeutically in inflammatory skin diseases and lymphoma.

As mentioned above for sMZL, WGS was performed by da Silva Almeida et al. [10] on normal and tumor samples of 25 Sézary syndrome (SS) and 17 other cutaneous (c) T-NHL patients. They present a distinctive pattern of somatic copy number alterations in SS, including highly prevalent chromosomal deletions involving the TP53, RB1, PTEN, DNMT3A, and CDKN1B tumor suppressors. Mutation analysis identified a broad spectrum of somatic mutations in key genes involved in epigenetic regulation (TET2, CREBBP, KMT2D (MLL2), KMT2C (MLL3), BRD9, SMARCA4, and CHD3) and signaling, including MAPK1, BRAF, CARD11, and PRKG1 mutations driving increased MAPK, NF-κB, and NFAT activity upon T cell receptor stimulation. The authors conclude that their findings provide new insights into the genetics of SS and cT-NHL and support the development of personalized therapies targeting key oncogenically activated signaling pathways for the treatment of these diseases. However, for me, this study, like many other similar studies, provide a large number of genetic changes in certain tumors, but provide actually quite little insight and reveal no unknown target for new drugs.

Ohtani et al. [11] give further insight in the pathogenesis of angioimmunoblastic T-NHL (AITL). In AITL, there is expression of CXCL13 which is used as a tumor marker. However, it is not clear whether the CXCL13(+) cells are the neoplastic TFH cells or follicular dendritic cells (FDCs). Using double-labeling immunofluorescent microscopy, they first showed that CXCL13 was mainly expressed in FDCs of normal germinal centers. In 28 of 33 AITL cases, CXCL13 was also mainly expressed in FDCs as a meshwork pattern but in the other five cases, CXCL13 was expressed in neoplastic cells. Triple-labeling immunofluorescent microscopy showed that the CXCL13(+) FDC meshwork in AITL harbored both neoplastic cells and B cells. CXCR5, the receptor of CXCL13, was expressed in neoplastic cells in AITL. These data indicate that neoplastic cells in some cases of AITL preserve a certain level of TFH-cell function since neoplastic cells and B cells are closely enmeshed in the CXCL13(+) cell-rich FDC meshwork in a similar way as in normal germinal centers.

Nasal natural killer T cell lymphoma (NKTL) is a highly malignant tumor that is closely associated with Epstein-Barr virus (EBV) infection. Latent membrane protein 1 (LMP1) is encoded by EBV and plays an important role in EBV-induced cell transformation. Therefore, Sun et al. [12] assessed the function of LMP1 as a stimulant of NKTL progression and the underlying mechanism. A human EBV-positive NKTL cell line (SNK-6) was transfected with pcDNA3.1-LMP1, LV-LMP1 shRNA, or LV-eukaryotic translation initiation factor 4E (eIF4E)-shRNA. Their results showed that LMP1 was highly expressed in SNK-6 cells compared with control groups. Following pretreatment with LMP1 shRNA, the proliferation of SNK-6 cells was inhibited and resulted in a G0/G1 phase arrest. A reduction in invasion and migration was also observed. LMP1 silencing promoted cell apoptosis. Further mechanistic analysis suggested that LMP1 overexpression induced the expression of eIF4E, while eIF4E-shRNA dramatically attenuated the increase in cell proliferation, invasion, migration, and the inhibition of apoptosis triggered by LMP-1 upregulation. Moreover, the effect of LMP1 on eIF4E expression was mediated by the NF-κB pathway.

## Epidemiology of lymphoma

The genetic susceptibility for the development of disease can only be unraveled when the disease is well defined. Delahaye-Sourdeix et al. [13] provide a fine example, by showing that a polymorphism close to a *HLA* gene is associated with Epstein-Barr virus (EBV)-positive HL (both mixed and nodular sclerosis type) but not with EBV negative HL. They could do this since they had a large series of cases (1200), which was compared with 5726 controls. The results were confirmed in an independent series of 468 cases and 551 controls.

De Montpréville et al. [14] describe their experience with 16 cases of lymphoproliferative disorders (PTLD) after lung transplantation. Their patients (15–63 years of age) were mostly (12/16) EBV seropositive at the time of transplantation. Eleven cases, occurring within 18 months after transplantation, had EBV+ DLBCL. Lungs and/or thoracic lymph nodes were often involved (*n* = 8). Two patients are in complete remission at 26 and 216 months. Nine patients died 8.0 ± 6.5 months after diagnosis. Of the five cases with late PTLD occurring 4–23 years after transplantation, one had pulmonary lymphomatoid granulomatosis (the only endothoracic case), one cutaneous large T cell lymphoma, two had anaplastic large cell lymphomas, and one HL. Two of the five cases were EBV-negative, including one followed by a second EBV + positive PTLD after 8 years of complete remission. Two patients were alive and well (follow-up: 44 and 151 months). These data are in line with the suggestion that early PTLD is generally an immune deficiency and EBV-associated disease, but that late lesions have a different and variable pathogenesis.

## Defining entities

### Hodgkin lymphoma

The distinction between extensive progressive transformed germinal centers (PTGC) and nodular lymphocyte predominant (NLP) HL is not easy. The presence of regular large CD20-positive cells has been described as indicative of NLPHL, but in practice, I prefer the criterium that the lesions need to have a mass effect, a disturbance of the lymph node architecture. Hartmann et al. [15] describe the morphologic features of 160 cases of PTGC for better delineation of PTGC from early involvement by NLPHL (although I do not know what early involvement really means… it is in my mind NLPHL or not). They had 93 patients with PTGC who never developed a lymphoma, 23 patients with synchronous PTGC and NLPHL, and 44 patients with PTGC with antecedent or subsequent history of lymphoma. The authors describe five patterns of PTGC that reflected progressive dismantling of germinal centers and here was no difference in the distribution of patterns 1 to 4 among the three groups of PTGC; however, in patients showing synchronous involvement of PTGC and NLPHL, pattern 5, which resembles a naïve B cell follicle, was significantly more frequent. The problem with this study and similar ones is a common problem in pathology: we do not have a gold standard, so we tend to formulate criteria based on circular argumentation.

A typical feature of NLPHL is the increased number of CD57-positive CD4+ T cells [16], cells that are normally present in the germinal center of lymphoid follicles. They show that the cells directly rosetting LP cells are positive for CD57 and/or for two markers of T-follicular helper (TFH) cells, PD-1, and BCL-6. Furthermore, by flow cytometry, more than 90 % of CD57+ T cells express Bcl6 and PD-1, whereas about half of the PD-1+ T cells are CD57+, both in normal tonsil and in NLPHL.

Eladl et al. [17] describe the clinicopathologic features of 25 cases of NLPHL and point to another feature: the presence of macrophages. The median age at onset of their patients was 56 years (range: 6–82 years) with male predominance (64 %). All patients presented with lymph node enlargement with predilection for cervical LNs. Seven cases (28 %) had a mediastinal lesion, and four (16 %) had extranodal involvement. Most cases (76 %) presented with early clinical stages. After median follow-up of 44 months, both of overall and progression-free survival rates were 95 %. The presence of >5 % CD68+ cells in NLPHL was significantly associated with older age at diagnosis and lower CR rate after initial treatment (42.9 vs 91.7 %). The presence of >5 % CD163+ cells was significantly correlated with presence of B symptoms (40 vs 0 %). However, there was no impact on overall survival of the presence of CD68- or CD163-positive macrophages.

Classical (c) HL is defined by morphology and aberrant B cell program. Rare cases of otherwise typical HL have strong CD20 expression. Benharrough et al. [18] describe 24 (13 % of their 166 cHL cases, which seems to me quite high) such cases. Except for an older age and a lower expression of CD15, no differences with CD20-negative HL were found, indicating that CD20 expression may be accepted in the diagnosis of cHL. I am not sure whether high CD20 and low CD15 is really acceptable for a HL diagnosis for me; these remain cases with important differential diagnosis of EBV-related disease or so-called gray zone cases in a majority of HRS cells in cHL.

### B cell lymphomas

Nodal marginal zone lymphoma (NMZL) remains an ill-recognized entity that easily may be misdiagnosed as FL. In this issue of the Journal of Hematopathology, van den Brand et al. [19] hypothesize that many translocation t(14;18)-negative FL may actually be NMZL. In another study of the same author, he investigates whether this mistake is clinically relevant [20]. The clinical features of 56 patients with NMZL were compared to 46 patients with FL. Both patient groups had a largely similar clinical presentation, but patients with FL had a higher stage at presentation, more frequent abdominal lymphadenopathy and bone marrow involvement, and showed more common transformation into diffuse large B cell lymphoma (DLBCL) during the course of their disease. Overall survival and event-free survival were similar for patients with NMZL and FL, but factors associated with worse prognosis differed between the two groups. Transformation into DLBCL was associated with a significantly poorer outcome in both the groups, but the phenotypes were different: DLBCL arising in FL was mainly of GBC phenotype, whereas DLBCL arising in NMZL was mainly of ABC phenotype, well fitting with the findings mentioned earlier in this review. Conconi et al. [21] also analyzed transformation in 340 MZL patients diagnosed and treated between 1995 and 2012: 157 ENMZL, 85 SMZL, and 37 nodal NMZL. With a median follow-up of 4.8 years, the median overall survival and progression-free survival of the whole population were 14.5 and 5 years, respectively. Transformation was observed in 13 cases (3.8 %; 5 % of SMZL, 4 % of ENMZL, 3 % of NMZL) indicating that the risk of transformation across all MZL types is lower than in FL, which is in contrast with the mentioned study into NMZL [20].

Choung et al. [22] investigated the presence of t(11;18) API2-MALT1, t(14;18) IgH-MALT1 translocations and chromosomes 3 and 18 aneuploidies in 30 ocular MZL, using fluorescence in situ hybridization (FISH); the t(14;18) IgH-MALT1 translocation was demonstrated in only one case, and the t(11;18) API2-MALT1 translocation was not found; trisomy 3 was observed in three cases, and five cases showed trisomy 18. The translocation-positive case also showed trisomy 18. There were no statistically significant correlations between chromosomal aberrations and clinical characteristics and treatment responses.

Lymphoplasmacytic lymphoma (LL) secreting IgA or IgG is rare. Cao et al. [23] evaluated 17 such patients, with IgA in eight and IgG in nine. The IgA-LPL group was more likely to present with B symptoms, a high beta2-microglobulin level and extramedullary involvement. Compared with patients with IgM-positive LL, there were similar clinical and pathologic features, but a higher mortality within the first year after diagnosis and worse overall survival.

MYD88 mutation is a hallmark of LL, but is also described in other lymphomas, including primary testicular DLBCL. Oishi et al. [24] investigated the mutational status in 23 cases of testicular DLBCL. There were 17 cases of primary testicular DLBCL and 6 secondary: 82 % (14/17) and 80 % (4/5) had a MYD88 mutation. The MYD88 mutational status nor the expression pattern of the protein affected overall survival, but it must be stated that only few cases lacked the translocation.

Mantle cell lymphoma (MCL), characterized by the t(11;14) (q13;q32) and cyclin D1 overexpression, commonly has overexpression of SOX11. Silencing of SOX11 in MCL cells promotes the shift into an early plasmacytic differentiation phenotype. Ribera-Cortada et al. [25] correlated the terminal B cell differentiation phenotype in 60 MCL with SOX11-expression: monotypic plasma cells and lymphoid cells with plasmacytic differentiation expressing cyclin D1 were observed in 7 (37 %) SOX11-negative but in none of 41 SOX11-positive MCL. Furthermore, BLIMP1 and XBP1 expression was also significantly more frequent in SOX11-negative than that in -positive cases (83 vs 34 % and 75 vs 11 %, respectively). However, no differences in the expression of IRF4/MUM1 were observed. The authors conclude that their results indicate that SOX11-negative MCL may be a particular subtype of this tumor characterized by more frequent morphological and immunophenotypic terminal B cell differentiation features that may be facilitated by the absence of SOX11 transcription factor. A bit weird conclusion, since basically they merely describe a feature within a well-defined entity without further arguments that this feature might indicate a subtype.

A bit better defined variant of MCL is the blastoid subtype, although there is still substantial interobserver variation in recognition of the morphological feature. Bhatt et al. [26] analyzed a series of 169 cases of MCL for the overall survival of different subtypes. At 5 years, blastoid and diffuse subtypes had worse survival compared to nodular subtype. However, the use of stem cell transplantation was associated with lower risk of death.

Mottok et al. [27] analyzed 45 primary mediastinal B cell lymphomas (PMBCL) and 3 PMBCL-derived cell lines for the presence of genetic alterations involving the major histocompatibility complex (MHC) class II transactivator CIITA and found frequent aberrations consisting of structural genomic rearrangements, missense, nonsense, and frame-shift mutations (53 % of primary tumor biopsies and all cell lines). They also detected intron 1 mutations in 47 % of the cases, and detailed sequence analysis strongly suggests AID-mediated aberrant somatic hypermutation as the mutational mechanism. Furthermore, they demonstrate that genomic lesions in CIITA result in decreased protein expression and reduction of MHC class II surface expression, creating an immune privilege phenotype in PMBCL.

Endemic (e) BL is found in children in equatorial regions and represents the first historical example of a virus-associated human malignancy. EBV infection and MYC translocations are hallmarks of the disease; it is unclear whether other factors may contribute to its development. Abate et al. [28] analyzed 20 eBL cases from Uganda and showed that the mutational and viral landscape of eBL was more complex than previously reported: other herpesviridae family members were present in eight cases (40 %), in particular human herpesvirus 5 and human herpesvirus 8; there was a distinct latency program in EBV involving lytic genes in association with TCF3 activity; there were lower frequencies of mutations in MYC, ID3, TCF3, and TP53, and a higher frequency of mutation in ARID1A; recurrent mutations in two genes not previously associated with eBL were identified in 20 % of tumors: RHOA and cyclin F (CCNF).

Some cases of morphological and phenotypical characteristic BL lack a MYC translocation. De Falco et al. [29] compared MYC translocation-positive and -negative BL and found four microRNAs differentially expressed between the two groups. In MYC translocation-negative cases, they found overexpression of DNA-methyl transferase family members, consistent to hypo-expression of the hsa-miR-29 family. This finding suggests an alternative way for the activation of lymphomagenesis in these cases, based on global changes in methylation landscape, aberrant DNA hypermethylation, lack of epigenetic control on transcription of targeted genes, and increase of genomic instability. In addition, they observed an overexpression of another MYC family gene member, MYCN that may therefore represent a cooperating mechanism of MYC in driving the malignant transformation in those cases lacking an identifiable MYC translocation but expressing the gene at the mRNA and protein levels.

### T cell lymphomas

Xue et al. [30] describe their experience with 225 patients with mature T cell malignancy, including 29 cases of T cell lymphoproliferative disorders (T-LPD, all with BM infiltration) and 196 cases of T-/natural killer-cell lymphoma (T/NKCL, 56 with BM infiltration and 140 without BM infiltration), figures clearly showing the different epidemiology in Western countries. The estimated 5-year overall survival rates of T-LPD and T/NKCL were 97 and 37 %, respectively. T/NKCL patients with BM infiltration showed significantly lower response rates and shorter survival than those without BM infiltration but clinical characteristics were more useful in effectively stratifying these patients.

Scarfo et al. [31] used advanced bioinformatics on a gene-expression dataset of 249 cases of T-NHL and normal T cells. Ectopic coexpression of ERBB4 and COL29A1 genes was detected in 24 % of ALK-negative anaplastic large cell lymphoma (ALCL) patients. ERBB4 expression was confirmed at the protein level by western blot analysis and immunohistochemistry. They also demonstrated that ERBB4-truncated forms show oncogenic potentials and that ERBB4 pharmacologic inhibition partially controls ALCL cell growth and disease progression in an ERBB4-positive patient-derived tumorgraft model. This elegant study identified a new subclass of ALK-negative ALCL characterized by aberrant expression of ERBB4, which might potentially be a therapeutic target.

Menon et al. [32] report the clinical, morphologic, immunophenotypic, and molecular characteristics of 18 cases of the rare primary central nervous system T-NHL (PCNSTL). Fifteen cases were classified as peripheral T-NHL, not otherwise specified, two of which were of γδ T cell derivation and one was TCR silent; there was one ALCL, ALK-positive and two ALCL, ALK-negative. The median age of the patients was 58.5 years (range, 21 to 81 years), with an M:F ratio of 11:7. Regardless of subtype, necrosis and perivascular cuffing of tumor cells were frequently observed (11/18 cases). CD3 was positive in all cases but one; 10/17 were CD8-positive, and 5/17 were CD4-positive. Most cases had a cytotoxic phenotype with expression of TIA1 (13/15) and granzyme-B (9/13). Polymerase chain reaction analysis of T cell receptor γ rearrangement confirmed a T cell clone in all 14 cases with adequate DNA quality. Next-generation sequencing showed somatic mutations in 36 % of cases studied; two had >1 mutation, and none showed overlapping mutations. These included mutations in *DNMT3A*, *KRAS*, *JAK3*, *STAT3*, *STAT5B*, *GNB1*, and *TET2* genes, genes implicated previously in other T cell neoplasms. The outcome was heterogenous: two patients are alive without disease, four are alive with disease, and six died of disease. In conclusion, PCNSTLs are histologically and genomically heterogenous with frequent phenotypic aberrancy and a cytotoxic phenotype in most cases.

Aggerwal et al. [33] encountered a case of S100-positive T-PLL and decided to study more cases: 19 additional T-PLLs and 56 other T cell lymphomas that are usually CD4-positive, including 15 AITL, 24 ALCL, 7 mycosis fungoides/SS, and 10 T-NHL NOS. Thirty percent (6/20) of T-PLLs were S100-positive, compared with 0/56 other T cell lymphomas. There were no significant differences between the S100 and S100 T-PLLs with regard to the male:female ratio (2:1 vs 1:1), age (72 vs 65), peripheral blood lymphocyte count (68 vs 101 × 10/L), or median survival (463 vs 578 days, where known). These results may have some diagnostic implication, but there were no clinical differences between the S100 and S100 T-PLLs.

## New entities/subtypes

Now that more and more knowledge on IgG4-related disease is accumulating, retrospective studies in different organs reveal relevant information. Ferry et al. [34] reviewed 38 cases presenting with inflammatory lesions in the orbita, previously diagnosed as orbital inflammatory pseudotumor or chronic dacryoadenitis. From these, 15 fulfilled the criteria for IgG4-related disease, nine men and six women, aged 24 to 77 years (median, 64 years); eight presented in the orbital soft tissue, six in the lacrimal gland, and one in the canthus; in one case, a clonal IGH gene rearrangement was detected; four had also disease in other anatomic sites. There were five patients, one man and four women, aged 26 to 74 years (median 50 years) who had orbital lesions (two involving lacrimal gland, three involving soft tissue) suspicious for, but not diagnostic of, IgG4-related disease. No patient developed lymphoma, and no patient died of complications of IgG4-RD. The other 18 patients had a variety of diseases, including granulomatosis with polyangiitis (3 cases), Rosai-Dorfman disease (1 case), nonspecific chronic inflammation and fibrosis involving lacrimal gland or soft tissue (12 cases), and others. Careful evaluation of histologic and immunophenotypic features and clinical correlation are required to distinguish orbital IgG4-RD from other sclerosing inflammatory lesions in the orbit.

## Prognostic/predictive factors in lymphoma

Finding predictive markers for expensive drugs is considered a very important method to reduce health care costs, but is not easy task. This is examplified by the work of Kenkre et al. [35]. Based on pre-clinical studies that suggest that single nucleotide polymorphisms (SNPs) in the Fcγ receptor (*FCGR*) genes influence response to rituximab they prospectively obtained specimens of 408 previously untreated, low tumor burden FL patients treated with single agent rituximab. The response rate to initial rituximab was 71 % but no FCGR genotypes or grouping of genotypes were predictive of this response or response duration. Although this is a disappointing result, the material and data are unique, and may provide positive results in the future.

Another potential promising approach was chosen by Nelson et al. [36] who developed an automated method, the hypothesized interaction distribution (HID) analysis, to analyze spatial patterns of multiple biomarkers in tissues and applied that to tumor-infiltrating lymphocytes in FL. A tissue microarray of 40 patient samples was used for multispectral imaging to determine the numbers and locations of CD3(+), FOXP3/CD3(+), and CD69/CD3(+) T cells. Higher numbers of all three cell types was related to favorable prognosis. More importantly, HID analysis of cell pattern identified patient subgroups with statistically significantly different survival (36 vs 142 months): a diffuse pattern of FOXP3 and CD69-positive T cells was related to good prognosis. This approach indicates hat complex tissue evaluation may result in relevant predictive biomarkers from the tumor microenvironment which may especially be relevant for immunotherapy. However, more cases and relation to specific therapies are needed before such approaches can be introduced in routine practice.

Chuang et al. [37] took a more traditional approach: they analyzed known prognostic factors in 174 DLBCL patients who were older than 60 years. They found that neither immunoblastic morphology nor GCB/ABC subtype correlated with survival, but IPI ≥ 3, B symptoms, bone marrow/peripheral blood involvement, EBER positivity, and CD5 positivity did.

Copie-Bergman et al. [38] analyzed 774 DLBCL cases characterized for cell of origin by the Hans classifier using FISH with BCL2, BCL6, MYC, immunoglobulin (IG)K, and IGL break-apart and IGH/MYC, IGK/MYC, and IGL/MYC fusion probes. MYC-R was observed in 51/574 (8.9 %) evaluable DLBCL cases. MYC-R cases were predominantly of the GBC-subtype 37/51 (74 %) with no distinctive morphologic and phenotypic features. Nineteen cases were MYC single-hit, and 32 cases were MYC double-hit (MYC plus BCL2 and/or BCL6) DLBCL. MYC translocation partner was an IG gene in 24 cases (MYC-IG) and a non-IG gene (MYC-non-IG) in 26 of 50 evaluable cases. Noteworthy, MYC-IG patients had shorter overall survival compared with MYC-negative patients, whereas no survival difference was observed between MYC-non-IG and MYC-negative patients. These are very relevant findings indicating that the mere MYC rearrangement is not the optimal biomarker for stratifying patients for more aggressive therapy.

Miura et al. [39] tried to use a translation of DNA alterations into immunohistochemistry, although it is well known that there is no good correlation. From their 38 patients treated with a salvage treatment consisting of rituximab, ifosfamide, etoposide, cytarabine, and dexamethasone followed by consolidative high-dose chemotherapies, a total of 17 cases (45 %) had expression of both MYC and BCL2. This was associated with a lower overall response rate (35 vs 71 %), worse 2-year progression-free survival (9 vs 67 %) and overall survival (35 vs 71 %). These are large differences, and the findings are in line with other studies.

Okina et al. [40] investigated the prognostic relevance of expression of REV7, a multifunctional protein involved in DNA damage tolerance, cell-cycle regulation, gene expression, and carcinogenesis. They give as reason for their study that its expression is associated with poor prognosis in human solid tissue cancers, but that the significance of REV7 expression in hematopoietic malignancies is unclear. They analyzed 83 specimens of de novo DLBCL (38 GCB and 45 ABC) treated with rituximab, cyclophosphamide, doxorubicin, vincristine, and prednisolone and found aberrant REV7 expression to be associated with significantly shorter overall survival, which held up in a multivariate analysis.

These studies reveal once again that we have many prognostic factors, but there is still little use of them in clinical trials.

## Ancillary techniques

Detection of light chain restriction (LCR) in FFPE-tissue remains often not successful and is a potential help in some cases. Arora et al. [41] described a new method, branched-chain RNA (bRNA) ISH assay for immunoglobulin κ constant (IGKC) and immunoglobulin λ constant (IGLC). It is a bit irritating that they use “clonality” rather than “light chain restriction,” and it should be well known that these terms cannot be used interchangeably. They found LCR (light chain ratio > 10:1) in 22 of 28 cytology specimens with a final diagnosis of lymphoma. In two cases, a κ predominance was present, although the ratio was <10:1. Two B cell lymphomas lacked IGKC and IGLC, whereas two cases were negative for the HKG. In 12 of the 20 cases with reactive lymphoid tissue, bRNA ISH identified a polyclonal lymphoid population. No light chain messenger RNA was detected in six cases (typically those associated with very few B-cells). There was no comparison with the traditional approach, so it is not clear how much this method will add.

According to Kirsch et al. [42] early diagnosis of cutaneous T cell lymphoma (CTCL) is difficult and takes on average 6 years after presentation, in part because the clinical appearance and histopathology of CTCL can resemble that of benign inflammatory skin diseases. This is indeed my experience as well, but I am not sure whether patients are helped with an earlier diagnosis. This topic was actually well reviewed in the previous issue of the Journal of Hematopathology [43]. According to Kirsch et al. [42], detection of a malignant T cell clone is critical in making the diagnosis of CTCL, but the T cell receptor γ (TCRγ) polymerase chain reaction (PCR) analysis in current clinical use detects clones in only a subset of patients. This is clearly not correct; in fact, using the Euroclonality approach, virtually all lymphomas have a detectable clone [44]. The problem is actually that clones are present in benign T cell proliferations of the skin too [45]. Nevertheless, their approach is interesting. High-throughput TCR sequencing (HTS) detected T cell clones in 46 of 46 CTCL patients, which according to the authors was more sensitive and specific than TCRγ PCR, and successfully discriminated CTCL from benign inflammatory diseases. The number of patients in the study is however fairly low for such strong statements. Nevertheless, sequence information of antigen receptor genes in lymphoproliferations is certainly going to change the way we look into lymphomas as described in my previous review [46, 47].
